# Mitochondrial DNA: A Key Regulator of Anti-Microbial Innate Immunity

**DOI:** 10.3390/genes11010086

**Published:** 2020-01-11

**Authors:** Saima Kausar, Liqun Yang, Muhammad Nadeem Abbas, Xin Hu, Yongju Zhao, Yong Zhu, Hongjuan Cui

**Affiliations:** 1 State Key Laboratory of Silkworm Genome Biology, Key Laboratory for Sericulture Biology and Genetic Breeding, Ministry of Agriculture and Rural Affairs, College of Biotechnology Southwest University, Chongqing 400716, China; drkausarsn@hotmail.com (S.K.); cysylq@swu.edu.cn (L.Y.); huxinusing@163.com (X.H.); zhuy@swu.edu.cn (Y.Z.); 2 Cancer Center, Medical Research Institute, Southwest University, Chongqing 400716, China; 3 Chongqing Engineering and Technology Research Center for Silk Biomaterials and Regenerative Medicine, Southwest University, Chongqing 400716, China; 4 College of Animal and Technology, Southwest University, Chongqing 400716, China; zyongju@163.com

**Keywords:** innate immunity, interferon, microbial pathogens, mitochondrial DNA, stimulator of IFN genes

## Abstract

During the last few years, mitochondrial DNA has attained much attention as a modulator of immune responses. Due to common evolutionary origin, mitochondrial DNA shares various characteristic features with DNA of bacteria, as it consists of a remarkable number of unmethylated DNA as 2′-deoxyribose cytidine-phosphate-guanosine (CpG) islands. Due to this particular feature, mitochondrial DNA seems to be recognized as a pathogen-associated molecular pattern by the innate immune system. Under the normal physiological situation, mitochondrial DNA is enclosed in the double membrane structure of mitochondria. However, upon pathological conditions, it is usually released into the cytoplasm. Growing evidence suggests that this cytosolic mitochondrial DNA induces various innate immune signaling pathways involving NLRP3, toll-like receptor 9, and stimulator of interferon genes (STING) signaling, which participate in triggering downstream cascade and stimulating to produce effector molecules. Mitochondrial DNA is responsible for inflammatory diseases after stress and cellular damage. In addition, it is also involved in the anti-viral and anti-bacterial innate immunity. Thus, instead of entire mitochondrial importance in cellular metabolism and energy production, mitochondrial DNA seems to be essential in triggering innate anti-microbial immunity. Here, we describe existing knowledge on the involvement of mitochondrial DNA in the anti-microbial immunity by modulating the various immune signaling pathways.

## 1. Introduction

Mitochondria are subcellular organelles with highly dynamic properties and found in large numbers in all eukaryotic cells [1]. Mitochondria play a crucial biological role in cellular homeostasis. They contain oxidative phosphorylation machinery that enables aerobic ATP production, and various metabolic pathways, including urea cycles, β-oxidation of fatty acids, and the tricarboxylic acid. Moreover, mitochondria have important biosynthetic activities, regulate cellular calcium metabolism and signaling, control thermogenesis, produce most cellular reactive oxygen species, and serve as the doorkeeper of the cell for programmed cell death [2,3]. Growing evidence suggests that mitochondria are also implicated in various unconventional processes, such as immune and defense responses against microbial pathogens and other stressors. For example, mitochondrial damage-associated molecular patterns and reactive oxygen species can activate anti-bacterial and anti-viral innate immune responses in mammals [1,4,5]. 

Besides other subcellular organelles, mitochondrion comprises of its genetic material: mitochondrial DNA. Human mitochondrial DNA exists as a double-stranded molecule, encoding tRNAs and rRNAs as well as respiratory chain subunits. The mitochondrial DNA is highly similar to that of bacterial ancestors, consists of a circular loop, and comprises a remarkable number of unmethylated DNA as CpG islands [6,7,8]. Multiple lines of evidence indicate that mitochondrial DNA is implicated in various types of innate immune regulation through inducing immune signaling pathways or initiating pathologies. In recent years, the involvement of mitochondrial DNA in anti-microbial immunity has attracted the attention of researchers worldwide. Mitochondria usually exist in the form of a connected networks, which are found even around the heart myofibrils and skeletal muscle. However, the small spheroids fragmented off the mitochondrial network undergo mitophagy, which would perhaps be one way of exposure of mitochondrial DNA into the cytosol [9]. In this review, we address the release of mitochondrial DNA from mitochondria under the stress conditions, e.g., microbial infection and its interactions with the DNA sensors, including TLR9, cGAS, and inflammasome in a cell. We also describe the recent literature regarding the effect of microbial infection, particularly viral and bacterial infection, on the release of mitochondrial DNA as well as its impact on the subsequent signaling pathways to limit the microbial infection. Furthermore, this article highlights the various mechanisms that are activated following the microbial infection. Thus, this article will help understand the relationship between microbial infection and mitochondrial DNA release and, subsequently, immune responses to limit infection. 

## 2. Mitochondria and Innate Immune Signaling Following Microbial Infection 

Mitochondria are an essential component of eukaryotic cells, which is the powerhouse of a cell that provides energy for normal physiological functions. Mitochondria play crucial biological roles in different interconnecting anabolic and catabolic processes such as glycolysis, oxidative phosphorylation, fatty acid b-oxidation, and the tricarboxylic acid cycle [10,11,12]. In addition, mitochondria have also been shown to involve in cellular signaling networks that control the innate immunity and inflammatory responses, cell survival/death, and calcium signaling [13,14]. Mitochondria are also involved in the redox signaling as it produces H_2_O_2_ when the cells are deficient of oxygen to stimulate the expression of transcription factors (e.g., hypoxia-inducible factor) that are needed for metabolic adjustment under low level of oxygen. Later, several studies suggested that mitochondrial reactive oxygen species also regulate various signaling pathways (e.g., NF-κB), and this method appears to be a way of communication between the function of mitochondria and other cellular processes for the maintenance of homeostasis [15,16,17,18]. Growing evidence suggests that mitochondrial DNA regulates immunological functions by interacting and governing TLR9, inflammasome, and stimulator of interferon genes (STING) pathways. Here, we discuss the current knowledge on the interaction of mitochondrial DNA with the factors mentioned above and subsequent signaling cascade. 

### 2.1. Mitochondrial DNA and Toll-Like Receptor 9

Toll-like receptor 9 (TLR9) is a protein receptor, which is encoded by the *TLR9* gene. This protein receptor is transcribed in different kinds of immune cells (e.g., macrophages, dendritic cells, etc.). Further, it has been shown that TLR9 is resided in the endoplasmic reticulum (ER) of immune cells and is translocated to the endosome upon detecting of unmethylated 2′-deoxyribose cytidine-phosphate-guanosine (CpG) DNA, a hallmark of microbial (e.g., bacterial) DNA [19,20]. Like the microbial genome, mitochondrial DNA also comprises of unmethylated CpG dinucleotides, the DNA act as a damage-associated molecular pattern that are endogenous molecules produced by cells undergoing abnormal cell death and are capable of stimulating the innate immune response in the host [21,22,23]. The class A, B, and C oligodeoxynucleotides (ODNs) are synthetic products (DNA nucleotides) comprising of unmethylated CpG nucleotide sequences like bacterial and mitochondrial DNA; however, they might have a different stimulatory effect on TLR9 activation [24]. Many studies reported that TLR9 senses different kinds of ODNs: class A ODNs stimulate plasmacytoid dendritic cells, while class B CpG ODNs trigger B cells [25,26]. Most of the studies demonstrated the interaction of mitochondrial DNA with TLR9 using class A ODNs. Following bacterial infection or leakage of mitochondrial DNA, TLR9 senses CpG DNA and drives immune responses [27,28] by activating downstream signaling cascades such as leucine-rich repeat pyrin domain containing 3 (NLRP3) inflammasomes, interferon regulatory factor (IRF)-dependent type 1 IFN, and pro-inflammatory nuclear factor kappa B (NFκB) [29].

There are several lines of evidence suggesting the deletion of the TLR9 gene has a positive impact on the health of animals as observed in various tissue injuries models, implying the association of TLR9 and mitochondrial DNA. For example, TLR9−/− mice improved survival outcomes in a necrotic lung model of cationic nanocarrier triggered necrosis and mitochondrial DNA leakage. The authors further reported the mitochondrial DNA induced pulmonary inflammation is remarkably decreased in MyD88−/− and TLR9−/− mice, suggesting the significance of the TLR9–MyD88 signaling [30]. Another study showed that intravenous administration of mitochondrial debris containing substantial amounts of mitochondrial DNA stimulated a systemic inflammatory response in wild-type mice, which was remarkably reduced in TLR9−/− mice [31]. Further, several independent studies under different experimental conditions demonstrated that lower concentration of circulating mitochondrial DNA has a better survival impact on the TLR9−/− mice [32,33,34]. Additionally, a study using a murine model of non-alcoholic steatohepatitis (NASH), also suggested that mitochondrial DNA from NASH hepatocytes caused a greater TLR9 activation compared with the control livers [35]. This data indicates the association of mitochondrial DNA with TLR9. This interaction not only activates the innate immune system but also augments the ensuing inflammatory response. Furthermore, the expression level of TLR9 seems to be related to the level of cytosolic mitochondrial DNA, suggesting the increased level of mitochondrial DNA release induces the high expression of TLR9 [27].

Currently, the interaction between mitochondrial DNA-TLR9 and subsequent signaling pathways activation has also been broadly studied in neutrophils. Mitochondrial DNA activates p38 mitogen-activated protein kinase (MAPK) by TLR9 with leakage of matrix metalloproteinase 8 and 9 in neutrophils [36]. Later, another study observed the increase of MMP9 and phosphorylated p38 in response to mitochondrial DNA treatment of neutrophils [37]. In addition, Wei et al. [30] also noted similar observation: pretreatment with ODN2088 (a TLR9 inhibitor) prevented p38 MAPK pathway activation and MMP8 leakage. Another study suggested that intratracheal injection of mitochondrial DNA can induce lung inflammation by TLR9–p38 MAPK pathway [38]. Concurrently, Gan and his co-workers [39] reported that Hip fracture in rats caused leakage of mitochondrial DNA and also greater activation of TLR9 and NFκB p65 result in lung injury. A recent study showed that leakage of mitochondrial DNA during lung ischemia-reperfusion induce TLR9-dependent neutrophil extracellular trap formation and drives lung injury [40]. Furthermore, ascites mitochondrial DNA correlated with worse progression free survival and the probability of disease progression. Mitochondrial DNA in ascites activates platelet and neutrophil responses that facilitate metastasis and block anti-tumor immunity [41]. The functional role of other MAPKs, including extracellular signal-regulated kinases and c-Jun N-terminal kinases, remains unclear. Overall, from these studies, it is evident that mitochondrial DNA can activate neutrophils by TLR9 binding and induction of the MAPK signaling pathway with subsequent production of MMP8 and MMP9.

### 2.2. Mechanism of Mitochondrial DNA Internalization and to Interact with TLR9

Mitochondrial DNA is located in the double membrane structure of mitochondria, to interact with TLR9, it must be either displaced from the entire mitochondria and translocated into the cell cytoplasm or, when extracellular, internalized through using some mechanism to interact with the endosomal TLR9 receptor [42]. To date, it is unclear how extracellular mitochondrial DNA is internalized, however, including phagocytosis, endocytosis, receptor-mediated endocytosis, and transmembrane diffusion are viewed as possible mechanisms for mitochondrial DNA internalization [43,44]. The transmembrane diffusion mechanism is seeming not appropriate for this internalization process as DNA is highly negatively charged, which makes it difficult to pass through the charged pores of the cell membrane. Another study suggested phagocytosis could be a suitable mechanism, as macrophages derived from monocytes can carry whole mitochondria leaked from necroptosis [45]. Further, many other studies also have shown that mitochondrial DNA interaction with additional cofactors, such as high-mobility group box 1 and receptor for advanced glycation end products, facilitates the internalization process into immune cells. High-mobility group box 1–CpG (class A) complexes cause TLR9/RAGE association and MyD88 recruitment in B cells [46,47]. However, this possible biological mechanism needs to be further evaluated. Viglianti and his co-workers [48] also proposed another mechanism for mitochondrial DNA internalization. They suggested that autoreactive B cells stimulation by mitochondrial DNA (CpG DNA) occurs following B cell receptor engagement, leading to the transfer of mitochondrial DNA to endosomal TLR9.

### 2.3. Mitochondrial DNA as a Stimulator of Interferon Signaling

The stimulator of interferon signaling (STING) is a cytoplasmic DNA sensor protein anchored in the endoplasmic reticulum. The STING pathway has been shown to mediate protective immune defense against infection through a wide range of DNA comprising microbial pathogens. The mitochondrial DNA is similar to that of microbial DNA, and has attained great attention as a mediator of innate immunity via the STING pathway [49,50]. STING protein is activated by direct interaction with cytosolic DNA, which is usually derived from intracellular viruses or bacteria or cyclic GMP-AMP synthetase and so on. Following activation, it induces interferon regulatory factor 3 that translocates to the cell nucleus and stimulates the transcription of type I interferons genes and also activates the NFκB signaling pathway [51,52].

Increasing evidence has shown that mitochondrial DNA is a crucial factor in activating the STING mediated IFN response [53]. The deficiency of apoptotic caspases (e.g., 3, 7, and 9) are responsible for the enhancement of type I IFN genes transcription. This biological phenomenon is associated with Bak-Bax; proapoptotic proteins belonging to the BCL2 family. These proteins are involved in the permeabilization of the outer membrane of mitochondria and promote the leakage of proteins associated with intermembrane space. Furthermore, they are involved in the leakage of mitochondrial DNA, and also the leakage of cytochrome C, which induces the intrinsic apoptotic pathway. Generally, apoptosis does not stimulate an inflammatory response. However, it has been shown that, when apoptotic process related caspases are suppressed, mitochondrial DNA goes on to induce cGAS-STING-mediated type I IFN signaling [54,55,56]. 

### 2.4. Mitochondrial DNA and the Inflammasome 

The inflammasome is the cytoplasmic multiprotein oligomers of the innate immune system that are involved in the activation of inflammatory responses. They are also the direct targets of mitochondrial DNA, leading to activation of caspase-1 and maturation of downstream factors such as interleukin-18 (IL-18) and IL-1β. So far, six different types of inflammasomes have been described: NLRP1, NLRP3, NLRP6, NLRP12, AIM-2, and NLRC4-IPAF [57]. Of these the inflammasomes, NLRP3 has been well studied. Nakahira et al. [57] suggested that suppression of autophagic protein enhances the level of cytosolic mitochondrial DNA in response to ATP and LPS in macrophages. Furthermore, the leakage of mitochondrial DNA is mediated by mitochondrial-derived reactive oxygen species, which might be related to the concentration of the NLRP3 inflammasome (also named as NALP3). This leakage of mitochondrial DNA is responsible for the secretion of IL-18 and IL-1β in response to LPS and ATP. It has been shown that the induction of mitochondrial reactive oxygen species correlates with higher production of active IL-1β in a caspase-1and NLRP3-dependent manner and treatment with mitochondrial reactive oxygen species scavengers suppresses this effect [58]. Several studies independently demonstrated the requirement of mitochondrial-derived reactive oxygen species in the activation of NLRP3 [59,60] and might be explained by its oxidizing influence on mitochondrial DNA. Recently Shimada and his co-workers [61] suggested that it is the oxidized form of mitochondrial DNA that confers the inflammatogenic potential to mitochondrial DNA. Mitochondrial reactive oxygen species trigger both the oxidative process and the cytosolic translocation of oxidized mitochondrial DNA that then binds directly to NLRP3 [61]. Further, it has been shown that non-oxidized mitochondrial DNA is incapable of activating the NLRP3 i, although it may induce IL-1β synthesis by other inflammasomes [62]. Genetic depletion of caspase-1 and NLRP3 causes less mitochondrial DNA leakage [57,60]. A previous study explored the biological mechanism by which mitochondrial DNA translocation is enhanced under the mitochondrial stress conditions, e.g., mitochondrial reactive oxygen species. The mitochondrial stress elicits the apoptosis process, Bcl2 (BAK), and its BAX stimulates mitochondrial outer membrane permeabilization, leading to the release of cytochrome c and apoptotic caspases activation. During this biological process, the network of mitochondria cooperates, and newly appeared BAK/BAX macropores allow components of the mitochondrial matrix, including mitochondrial DNA, leak into the cell cytosol [63]. The translocation of mitochondrial DNA also relies on the activation of NALP3 inflammasome, which finally triggers the secretion of IL-18 and IL-1β [57]. Later, Shimada and his co-workers [61] (2012) proposed that the intrinsic NLRP3 characteristics might cause the crucial biological role of mitochondrial reactive oxygen species during the process of NLRP3-dependent cytosolic leakage of mitochondrial DNA. The NLRP3 mainly binds oxidized mitochondrial DNA and further stabilizes cytosolic mitochondrial DNA following leakage, this proposes a positive feedback loop in which NLRP3 inflammasome activation via oxidized mitochondrial DNA further enhances the leakage of mitochondrial DNA to stimulate innate immunity [64]. Furthermore, in murine macrophages, the release of mitochondrial DNA during cell death also provides a second signal, which integrates with an additional inflammatory signal (e.g., LPS) to induce the production of IL-1β and stimulate the NLRP3 inflammasome [61]. Although different molecular mechanisms (e.g., base excision repair, and mismatch repair pathway) have been shown to be involved in the repair of the oxidized mitochondrial DNA, however, our knowledge is still limited regarding the repair mechanisms in this organelle. Furthermore, in some pathological conditions (e.g., neurodegeneration) oxidative damage to the mitochondrial DNA is extensive. It might not be manageable by the repair mechanisms as well as possibly mitochondria do not have well developed repair systems like the nuclear repair system [65]. 

## 3. Mitochondrial DNA and Anti-Microbial Immunity 

**Figure 1:**
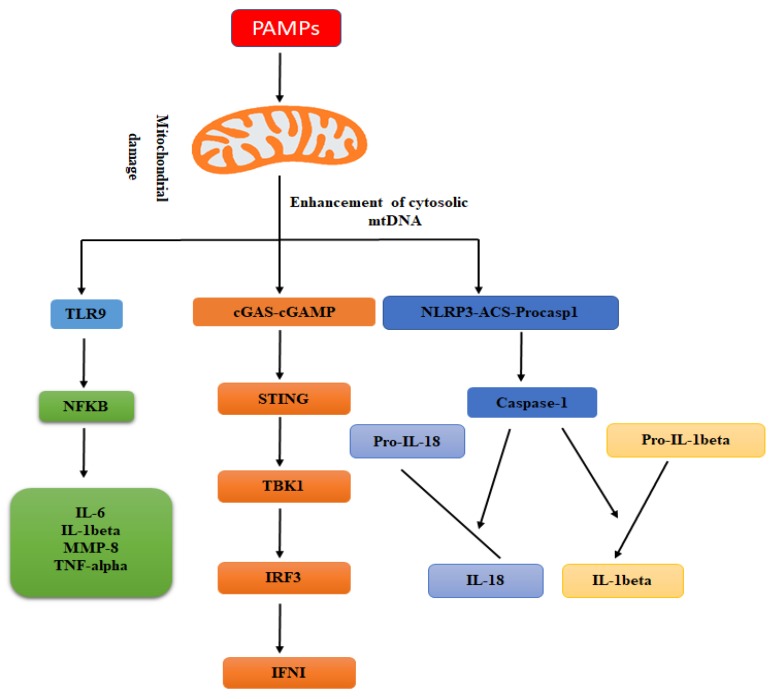
The schematic diagram is representing the molecular mechanism through which mitochondrial DNA stimulates innate anti-microbial immunity. Under the stress conditions, mitochondrial DNA is released from mitochondria that stimulate the production of pro-inflammatory cytokines (e.g., IL-6, IL-1β, MMP-8, and TNF-α,) and also enhance their release. The escaped mitochondrial DNA following the microbial infection is detected by the cGAS/stimulator of interferon genes (STING) signaling pathway that causes TBK1/IRF3 dependent production of type I interferon and also limits replication of microbes. However, the caspases, e.g., caspase-3, -7, and -9 activation regulates the intrinsic apoptotic signaling pathway. Furthermore, the escaped mitochondrial DNA after stress also stimulates the NLRP3 inflammasome to recruitment and activates caspase-1, which is involved in the cleavage of pro-IL-18 and pro-IL-1β into their active forms.

In animals, the immune system is furnished with an array of complementary molecular mechanisms to recognize different molecules related to microbial pathogens or with their effect(s) on the host cells to reduce the possibility for infectious microbial agents to evade detection of the immune system [66]. The DNA of mitochondrial is remarkably similar to that of bacterial DNA and has been shown to induce anti-microbial immunity [30]. In this section, we describe the functional role of mitochondrial DNA as a stimulator of anti-viral and anti-bacterial immunity (Figure 1). 

### 3.1. Mitochondrial DNA and Anti-Viral Immune Responses

Mitochondria are the critical organelles that supply energy for cell survival. Besides providing energy, they have been shown to modulate programmed cell death pathways and also control innate immunity [58]. Accumulating studies suggest that mitochondria machinery stimulates cellular inflammation, especially by eliciting anti-viral signaling pathways [4,67]. Viruses following infection produce pathogen associated molecular patterns, e.g., double stranded RNA. Many studies elucidated a biological role for IκB kinase-*i* and TBK1 as kinases crucial for phosphorylation and activation of IRF7 and IRF3 both in vitro and in vivo [68,69,70,71]. Later, it was shown that cytosolic RNA sensors, including melanoma differentiation-associated gene 5 (MDA5) and retinoic acid-inducible gene I (RIG-I), are essential to induce anti-viral immunity. These RNA sensors are capable of recognizing RNA containing viral species and stimulating signaling by mitochondrial-based anti-viral signaling that causes the transcription of cytokines (interferons: type I and III) to prevent replication of viruses [72].

Growing evidence suggests that in addition to mitochondria, mitochondrial DNA also participates in the anti-viral immunity. Mitochondrial DNA can be recognized as a cell-intrinsic elicit of anti-microbial (anti-viral) signaling, and mitochondrial DNA monitoring homeostasis collaborates with typical virus detecting mechanisms to entirely incite innate anti-viral innate immunity [73]. In this section, we describe the contribution of mitochondrial DNA in eliciting anti-viral immunity. 

The innate immune system of eukaryotes utilizes pattern recognition receptors to recognize PAMPs, e.g., viral nucleic acids. While RIG-I and TLR7 recognize viral RNA, e.g., influenza virus, the NLRP3 recognizes intracellular ionic fluxes after infection with influenza virus. The viroporins ion channel activity, such as EMCV 2B or M2 protein of influenza virus, is important for the activation of NLRP3 inflammasome [74,75]. It has been shown that viral infection results in the mitochondrial DNA leakage into the cytoplasm, which in turn stimulates anti-viral immunity. A recent study identified the mechanism by which Encephalomyocarditis virus and influenza virus induce mitochondrial DNA leakage into the cytosol by their viroporin (transmembrane pore-forming viral proteins) activity. The cytosolic translocation of mitochondrial DNA in response to Encephalomyocarditis virus or influenza virus infection induces cGAS- and DDX41-dependent innate immune responses. In addition, the STING pathway dependent anti-viral signaling is also amplified in neighboring cells by gap cellular junctions [55]. Given that the viroporin-stimulated disturbance in the intracellular ionic milieu is accompanied by Mn^2+^ efflux from membrane enclosed organelles, e.g., mitochondria and Golgi apparatus, the ion channel viroporins activity might be required for enhancing the sensitivity of cGAS to double stranded DNA. Following the infection of DNA viruses, cytosolic ion level is enhanced in the host cells, and this increase directly induces the activity of the cGAS/STING signaling pathway [76,77].

Another study suggested mitochondrial DNA stress as a cell-intrinsic induce anti-viral signaling and propose that cellular monitoring of mitochondrial DNA homeostasis collaborates with canonical virus detecting mechanisms to fully engage innate anti-viral immunity. For example, mitochondrial DNA stress induced by the virus, e.g., herpes viruses, result in strong anti-viral immunity. The authors demonstrated that moderate stress caused by mitochondrial DNA-binding protein of transcription factor A, mitochondrial (TFAM) deficiency involves cytosolic anti-viral signaling to enhance the production of a subset of interferon-induced genes. They further described that aberrant mitochondrial DNA packaging promotes the release of mitochondrial DNA into the cytosol, where it interacts with the cGAS and promotes STING-IRF3-dependent signaling to increase interferon-induced gene transcription, potentiate responses of type I interferon, and confer broad viral resistance. Moreover, this study suggested that herpes viruses elicit mitochondrial DNA stress, which promotes type I interferon responses and anti-viral signaling during infection [73]. This phenomenon has been elaborated by several studies, which suggest the significant enhancement of mitochondrial DNA in the cytoplasm of TF^D^ cells following stress conditions (e.g., microbial infection). Confocal and electron microscopy of these cells showed the presence of interconnected, elongated networks of mitochondria suggesting the mitochondrial hyperfusion since the fission of mitochondria facilitates the leakage of mitochondrial DNA into the cytosol [4,61,78]. The transduction of bone marrow-derived macrophage and mouse embryonic fibroblasts with replication incompetent retroviruses encoding the mitochondria targeted HSV-1 UL12 M185 gene product also showed the hyperfusion of mitochondria, enlargement of nucleoid, and loss of mitochondrial DNA. Furthermore, in these cells, herpes virus infection triggered the anti-viral immune signaling by inducing the expression of interferon stimulated gene, type I interferon, and phospho-TBK1 [73,79].

Furthermore, over the past decades, the dengue virus global incidence has dramatically increased, and it is now endemic in approximately more than 100 countries [80]. The longstanding model about cellular anti-viral molecular mechanisms in the setting of dengue virus infection focuses approximately exclusively on RNA sensors, including RIG-I and MDA5 [81,82]. In this paradigm, RNA viruses directly interact with RIG-I and trigger downstream IRF3 signaling, thereby enhancing IFN-I production [83,84]. Recent studies showed that cGAS has remarkable anti-viral properties against different positive-strand RNA viruses, including the Flavivirus family members. However, not negative-strand RNA viruses [85,86], suggesting that common characteristic features observed during the life cycle of positive strand RNA virus, including the massive rearrangement of internal membranes, may stimulate a mislocalization of DNA that could act as a substrate to induce the cGAS/STING signaling pathway [86]. For example, cGAS−/− mice displayed enhanced rates of infection and mortality when compared with wild type mice, which was infected with the +ssRNA West Nile virus. This infectious agent probably triggers the release of mitochondrial DNA into the cytosol, which is then recognized by the nucleic acid sensors in the cytoplasm, subsequently induce the immune system to clear the infection [86]. 

Dengue virus translates its polypeptide as one of the initial biochemical events upon entry into the target cell, dengue virus proteins rapidly colonize the endoplasmic reticulum membrane to make the scaffold essential for replication. The gathering of these viral proteins also reaches the mitochondrial membrane, which is nearby for calcium interchange with the lipid biosynthesis and endoplasmic reticulum [87]. Therefore, infection of the dengue virus profoundly influences this vital cellular organelle that is also an imperative hub for the innate immune signaling. It has been shown that the C-terminus of the dengue virus M protein targets the membrane of mitochondria, causing swelling of its matrix, permeabilization, and potential loss of mitochondrial membrane [88,89,90]. Furthermore, it has been suggested that the NS2B3 protease confined in the outer membrane of mitochondria and cleaves mitofusins-1 and -2, influencing the function and architecture of mitochondria upon the viral infection [91]. A recent study showed that cGAS could sense mitochondrial damage during infection of dengue virus to start type I IFN responses. In addition, mitochondrial DNA can elicit the stimulation of the IFN-β promoter by cGAS, acting as a danger associated molecular pattern leaked during infection of dengue virus [80,92]. Furthermore, the dengue virus utilizes active biological mechanisms to counteract the cGAS/STING signaling pathway by targeting two critical host proteins implicated in cytoplasmic RNA and DNA detection cGAS and STING [85,93]. Targeting the cGAS not only reduces the STING induction in the infected cell but also reduces the cGAMPs translocation to bystander cells through tight junctions [94].

Hepatocytes of non-alcoholic steatohepatitis patients have reported leakage of mitochondrial DNA in the form of microparticles [35], indicating the release of mitochondrial DNA through extracellular vesicles is an effective strategy for cellular homeostasis. Some virus infected cells have also been shown to form extracellular vesicles in large numbers and reported to be implicated anti-viral immune responses [95,96]. To date, various biological mechanisms have been reported regarding the formation of these extracellular vesicles after virus infection. For example, small Rab GTPase is shown to regulate the production of extracellular vesicles [97,98], and some of these proteins appear to be important modulators of the formation of extracellular vesicles in virus infected cells. The infection of cytomegalovirus enhanced the level of Rab27a that was associated with cytomegalovirus production [99]. Herpes simplex virus 1 also uses Rab27a protein for its transportation, intracellular transport, and exocytosis [100,101]. The interaction of Rab GTPase and virus regulates both the production of the virus and even the formation of the extracellular virus. The other mechanism proposed that the extracellular formation in cells infected with virus depends on the tetraspanin-dependent signaling pathways [102]. A recent study demonstrated that Herpes simplex virus 1 can induce the formation of CD63 positive extracellular vesicles and does not change the TSG101 exocytosis or Alix, indicating the viral infection elicits ESCRT-independent signaling pathways for the formation of extracellular vesicles [103]. Jeon et al. [104] suggested that the cGAS/STING signaling pathway is linked with the Kaposi’s Sarcoma-associated herpesvirus extracellular vesicles-mediated interferon-stimulated gene expressions, and mitochondrial DNA on the surface of Kaposi’s Sarcoma-associated herpesvirus extracellular vesicles is a causative factor. The authors further suggested that DNA-carrying extracellular vesicles from Kaposi’s Sarcoma-associated herpesvirus-infected cells could be a starting factor for the immune response against infection of the virus, which might be crucial to understanding the microenvironment of virus-infected cells. However, this study failed to completely resolve the mechanisms of Kaposi’s Sarcoma-associated herpesvirus extracellular vesicles mediated interferon-stimulated genes responses, other factors, and biological pathways that are associated with them. Many questions remained unanswered, firstly, whether the DNA inside the extracellular vesicles has the same effect as the DNA present on the extracellular vesicles. Secondly, the exact mechanisms of extracellular vesicles-mediated anti-viral response and their physiological significance in vivo also remain to be investigated.

The T cell interaction with antigen-bearing dendritic cells results in the activation of T cells and seems to have some physiological consequences on antigen-bearing dendritic cells function. A recent study demonstrated T cell antigen-bearing dendritic cells by the exosomal DNA transfer, supporting a particular function for antigen-dependent contacts in giving protection to antigen-bearing dendritic cells against infection of the pathogen. This study further provided evidence regarding the mechanism of transferring information from T cells to antigen-bearing dendritic cells. It indicates that after the interaction of these cells, signals are transmitted from the T cell to the antigen-bearing dendritic cells by extracellular vesicles that comprise mitochondrial and genomic DNA, to stimulate anti-viral immune responses by the cGAS/STING cytosolic DNA-recognizing signaling pathway and transcription of IRF3-dependent interferon modulated genes. This study further showed that extracellular vesicles-treated antigen-bearing dendritic cells are more resistant to subsequent viral infections, suggesting that this cellular interaction process can stimulate anti-viral immunity in antigen-bearing dendritic cells [105].

Interleukin-1 β: a proinflammatory cytokine, plays a vital biological role in various physiological processes, including immune responses, apoptosis inflammatory responses, cell cycle, and central nervous system. Multiple stimuli induce the expression of Interleukin-1 β; for example, its production is highly sensitive to microbial infection [106,107]. A recent study suggested that a novel function for Interleukin-1 β in the enhancement of cell-intrinsic immunity, with significant involvement for the cGAS/STING in integrating microbial and inflammatory cues for the host defense. The exogenous Interleukin-1 β stimulates the activation of interferon regulatory factor 3 in human fibroblast, myeloid, and epithelial cells. The activation of interferon regulatory factor 3 by Interleukin-1 β is dependent upon the adaptor of the DNA-recognizing signaling pathway, an inducer of interferon genes, by the sensation of cytosolic mitochondrial DNA through cGAS. To confirm whether the treatment of Interleukin-1 β can enhance the leakage of mitochondrial DNA, the authors performed immunoblast analysis using the cytosolic contents. They also analyzed the concentration of nuclear and cytosolic DNA fractions by quantifying the mitochondrial specific genes: MT-CO2, D-loop, MT-ATP6, and MT-ND1 and nuclear specific genes: *RPL13A.* These analyses confirmed the release of mitochondrial DNA following Interleukin-1 β administration. The authors proposed the increase of mitochondrial mass and membrane potential loss of mitochondria are the specific contributing factors in the leakage of mitochondrial DNA. Further, this study showed that Interleukin-1 β administration could enhance the production of interferon and interferon activation signaling to direct an effective innate immune response that limits dengue virus infection. [53].

### 3.2. Mitochondrial DNA and Anti-Bacterial Immunity

**Figure 2:**
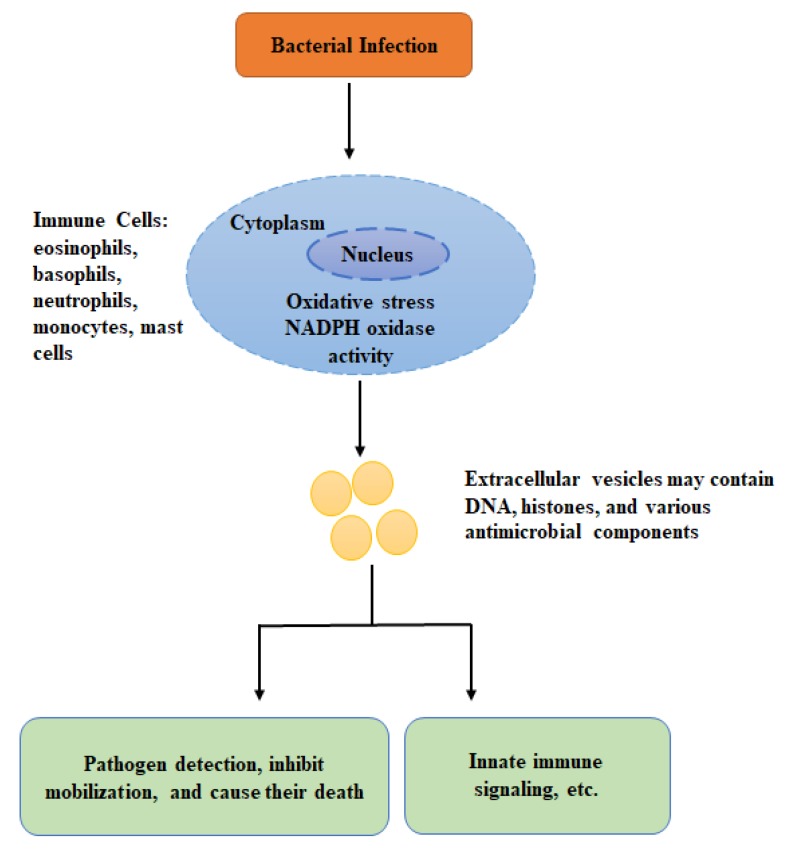
The schematic diagram is representing the effect of bacterial infection on immune cells that causes the formation of mitochondrial DNA-based extracellular vesicles to limit bacterial infection. The extracellular vesicles directly bind to the pathogens and causing their death. Furthermore, they are involved in the transportation of signals to the other immune cells, etc.

There are multiple lines of evidence suggesting the involvement of mitochondria in anti-bacterial immunity by producing reactive oxygen species. The mitochondrial derived reactive oxygen species plays a crucial biological role in macrophage related anti-bacterial immune responses [4]. Besides mitochondrial reactive oxygen species, mounting evidence suggests that mitochondrial DNA also contributes to anti-bacterial immunity. Due to common ancestors, DNA of mitochondria shares characteristic features with the bacterial genome, as mitochondrial DNA also consists of unmethylated DNA as CpG islands [6]. Due to this, it seems that mitochondrial DNA also acts as a pathogen-associated molecular pattern, and may induce the anti-bacterial immunity. This section focuses on the functional role of mitochondrial DNA in the stimulation of anti-bacterial immunity (Figure 2).

It has been shown after robust activation signals, various kinds of immune cells release granular proteins and chromatin into the cellular microenvironment (extracellular space), forming DNA-based extracellular traps. The process of extracellular traps formation is particularly prominent in neutrophils; however, it also exists in other immune cells, including basophils, eosinophils, mast cells, and macrophages. Mounting evidence suggests that extracellular traps are highly involved in the anti-bacterial and anti-fungal immune responses [108,109].

In human, host immune cells, eosinophils produce DNA-based extracellular traps (comprising of anti-microbial peptides, histones, and DNA) to capture and kill microbial pathogens [108]. Another study suggested that eosinophil can release mitochondrial DNA that acts as an extracellular trap. The mitochondrial origin of the leaked DNA was recognized by a combination of molecular biological and microscopic techniques [110]. The extracellular trap recognizes a microbial pathogen, restricts their mobilization, and is responsible for their death. Enzymatic degradation of extracellular DNA results in the incapability of eosinophils to kill bacterial pathogens in the extracellular space [98]. Therefore, although DNA in the extracellular traps is unlikely to be highly toxic for bacterial pathogens, it is indispensable for the anti-bacterial impacts mediated by eosinophils. The eosinophil extracellular traps process also depends on the production of reactive oxygen, like neutrophil and mast cells extracellular traps. However, unlike mast and neutrophils, the eosinophils do not commit suicide after leakage of mitochondrial DNA. Later, studies on mouse and human basophils suggested generation of reactive oxygen species and extra cellular traps comprising mitochondrial DNA upon IL-3 priming in the NADPH oxidase-independent form. Thus, it is believed that the stimulation of the NADPH oxidase is an essential intracellular signaling process for the formation of the extracellular traps. Genetic defects, as well as pharmacological inhibition of this NADPH oxidase, is associated with reduced generation of reactive oxygen species, eliminating the eosinophils ability to form extracellular traps. Although basophils lack phagocytosis activity, yet they can kill bacteria by extracellular traps that consist of granular proteins and mitochondrial DNA [111]. As described earlier, the pathogen-stimulated mitochondrial DNA-based extracellular vesicles in basophils and eosinophils have crucial biological roles in anti-bacterial immunity. Furthermore, a recent study suggested that eosinophils release extracellular DNA traps that are key extracellular structural components within eosinophil-rich secretions. Further, this study showed that eosinophil DNA traps entrapped not only bacteria but also fungal pathogens [112]. Besides eosinophils, neutrophils, monocytes, and mast cells have also been demonstrated to form extracellular traps containing DNA [113,114,115,116]. Interestingly, it has been shown that Panton Valentine toxin of *Staphylococcus aureus* can stimulate NADPH-oxidase-independent formation extracellular traps in neutrophils that produce nuclear blebs and released as extracellular traps [110,117]. However, there is controversy regarding the DNA source and its mechanism of leakage. Most of the researchers believe that the DNA in extracellular traps is of nuclear origin and needs cell death. In neutrophils, the kind of cell death leading to the formation of neutrophil extracellular DNA traps is termed as netosis [118]. However, for many reasons, a note of caution would be raised here: (1), it has been shown that neutrophil extracellular traps can be produced through viable neutrophils both in vivo [119] and in vitro [113,120]. (2) The formation of neutrophils extracellular traps happens much more rapidly compared to apoptosis, or any other type of programmed cell death utilizing stimuli known to slow down apoptosis [121]. (3) In most studies, the researchers had induced neutrophils with phorbol 12-myristate 13-acetate that is commonly known to stimulate fast necrosis comparable to cell death [122]. The DNA releasing out in the microenvironment (extracellular space) of phorbol 12-myristate 13-acetate induced neutrophils does not exhibit the typical DNA comprising of extracellular DNA traps fibers [123]. When typical neutrophils were described, it cannot be omitted that the structures were shaped before occurrence of cell death. (4) Cell death in association with neutrophils extracellular traps production does not necessarily show a novel kind of cell death. (5) It is hard to assume how a dead cell can release DNA since this process appears to require energy.

Besides, the abovementioned controversy about the DNA origin and its mechanism of leakage in neutrophils, the anti-bacterial role of the extracellular DNA structures have been studied mainly using neutrophils extracellular traps, contained histones, DNA, and numerous anti-microbial components, including cathepsin G, proteinase 3, neutrophil elastase, defensins, Bacterial Permeability-Increasing protein, and myeloperoxidase [124,125]. Neutrophils extracellular traps, like eosinophils extracellular traps, immobilize at high abundance of granule proteins capable of killing invading microbial pathogens [125]. Further evidence regarding the pathophysiological significance of extracellular DNA traps has also been observed in various microbial pathogens (e.g., bacteria), such as *Clostridium perfringens*, *Streptococcus pyogenes*, and *Streptococcus aureus* [126,127]. 

Invertebrates, like their vertebrate counterpart, also exhibit a similar phenomenon as DNA-based extracellular traps [128,129]. For example, a recent study on invertebrates, oyster *Crassostrea gigas* demonstrated that hemocytes of *Crassostrea gigas* produce and release anti-microbial H5-like and H1-like histones in response to damage of tissue and microbial infection that seem to have anti-microbial properties. These anti-microbial histones are also associated with extracellular networks of DNA leaked by hemocytes and assist them in the clearance of microbial pathogens. The hemocyte-leaked DNA was observed to surround and entangle vibrios. This immune defense biological mechanism is reminiscent of the extracellular traps of neutrophils recently reported in the oyster. Extracellular traps of the oyster have been reported in vivo in hemocyte-infiltrated interstitial tissues surrounding injuries, while the unchallenged tissues of the oysters do not contain these traps. The extracellular formation in oyster is highly dependent on the synthesis of reactive oxygen species from hemocytes, suggesting that extracellular formation depends on molecular and cellular mechanisms from invertebrates to vertebrates. Further, extracellular formation is an effective strategy control microbial infection in invertebrates [129].

*Streptococcus pneumoniae* is the most common bacterial pathogen, that is responsible for invasive pneumococcal diseases, and can produce considerable amounts of H_2_O_2_ [130,131,132]. Hamada and his co-workers [133] demonstrated that H_2_O_2_ could prevent cell migration and also impair airway epithelial cell repair. Additionally, A549 cells released lactate dehydrogenase (LDH) following exposure to H_2_O_2_ and also exhibited necrotic phenotype rather than programmed cell death [134]. A recent study suggested that *Streptococcus pneumoniae* secreted H_2_O_2_ causing damage of mitochondria and severe histopathological damage in lung tissue of mice. Due to the mitochondrial damage, mitochondrial DNA is released the cytoplasm, which subsequently stimulates the STING signaling pathway that stimulates the expression of type I interferons to limit the bacterial infection 6 [135]. Although, this study provided multiple lines of evidence regarding the activation STING signaling pathway and production of type I interferons and suggested that this signaling cascade might be involved in bacterial clearance; however, some further questions remained, such as whether this process favors the bacterial clearance or if it may aggravate host cell apoptosis.

Furthermore, pneumolysin, a pore-forming toxin is the key virulence factor of Gram-positive bacteria (*Streptococcus pneumoniae*) and is considered to influence alveolar epithelial cells to stimulate the immune system by the release of danger-associated molecular patterns [136,137]. Nerlich et al. [138] demonstrated that bacterially released pore-forming toxin pneumolysin could remarkably alter the ATP homeostasis of cells and lead to morphologic changes of mitochondria in alveolar epithelial cells of human in vitro. Furthermore, this toxin is also responsible for the influx of mitochondrial calcium and loss of mitochondrial membrane potential causing the opening of the permeability transition pore of mitochondria and leak of mitochondrial DNA via microvesicles without activation of intrinsic apoptosis. This liberation of mitochondrial DNA in response to *Streptococcus pneumoniae* strongly activates the innate immune system via STING signaling pathway for the clearance of bacterial pathogens.

Overall, under stress conditions, particularly during microbial infection, physiological changes occur in mitochondrial membranes leading to the leakage of mitochondrial DNA, which is an evolutionary beneficial biological mechanism in a host, amplifying anti-viral and anti-bacterial signaling in response to these pathogen invasions. However, the abnormal accumulation of damaged mitochondria and the release of mitochondrial DNA into the cytosol may also be responsible for various inflammatory diseases [130,132,139].

### 3.3. Mitochondrial DNA and Antifungal Immunity 

In nature, fungi are highly abundant and crucial to the environment. Among fungi, only a few species of them are pathogenic. They have long been identified as microbial pathogens of agricultural crops and result in widespread population declines of amphibians and bats, linked to both global movement of people and goods and environmental modification [140]. 

Hosts comprise a large variety of defense mechanisms against fungi and range from protective biological mechanisms that existed early in the evolution of multicellular living organisms (innate immunity) to sophisticated adaptive mechanisms that are stimulated particularly during pathogen infection [141]. Conventionally, innate immunity is a first line of defense, and it has received attention because, despite an absolute absence of specificity, it efficiently distinguishes self from non-self and stimulates adaptive immune mechanisms by the provision of specific signals [141,142]. The innate immune system confers quick recognition of microbial infection by a limited repertoire of germline-encoded receptors that sense a group of highly conserved molecular patterns common to wide-ranging groups of microbial species [143,144]. Once the fungi take entry into the body, they are encountered with a series of innate mechanisms of defense, such as cellular membranes, cellular receptors, and several humoral factors. In the tissues, phagocytes, comprising of neutrophils, mononuclear leukocytes (macrophages and monocytes), and dendritic cells, have an important role, and natural killer cells, γδ-T-cells, and non-hematopoietic cells, for example, endothelial and epithelial cells are implicated in the defense of the host [145]. 

*Candida albicans* is the most common fungal pathogen of humans, upon fungal infection host exploit various biological mechanisms to clear this pathogen. Following fungal infection, immune cells (e.g., granulocytes and monocytes) come into contact with the fungal pathogens, e.g., *Candida albicans*. Monocytes exert immediate anti-fungal activity and prevent germination, mediate phagocytosis, and kill fungal cells [146]. Furthermore, phagocytes synthesize calprotectin and lactoferrin proteins [147,148]. These proteins not only provide nutritional immunity (by preventing pathogens to essential nutrients), but are also involved in the formation of DNA-based extra-cellular traps [149,150]. Neutrophils surround the fungal hyphae (which are too large to phagocytose) and go through programmed cell death, leading to DNA decompensation and formation of extra-cellular traps. These extra-cellular traps capture and kill fungal pathogens [149,151]. A recent study reported that human monocytes also release DNA-based extracellular traps in response to *Candida albicans* and are involved in clearance without further inflammation. Generally, human monocytes spontaneously respond to *Candida albicans* cells via phagocytosis, and release DNA in the form of extracellular traps. The monocyte extracellular traps contain citrullinated histone, lactoferrin, myeloperoxidase, and elastase. Extracellular traps from activation complement and deposit C3b. However, factor H binds via C3b to the extracellular DNA, mediates cofactor activity, and prevents the induction of the inflammatory cytokine interleukin-1 β in monocytes [146]. Collectively, the biological role of mitochondrial DNA in anti-fungal immunity has not been largely studied, and future studies should focus on determining tge mechanism by which mitochondrial DNA improves the anti-fungal immunity.

## 4. Intercellular Transfer of Mitochondrial DNA 

Recently it has been shown that mitochondrial DNA is not constrained within cells; however, it can transfer between cells as reported in mouse models and tumor models of tissue damage [152,153]. A recent study suggested that mouse tumor cells lacking mitochondria grow tumors only after obtaining mitochondrial DNA from tumor cells in their microenvironment. The transfer of mitochondria between cells has also been reported after ischemia-stimulated injury in the brain, heart, and lung epithelium [154,155]. The ability of mitochondrial transfer between cells seems to be an evolutionarily conserved event related to diseases with the compromised mitochondrial role, including neuromuscular, neurodegenerative, and cardiovascular diseases, aging, and cancer [156]. 

So far, various biological mechanisms have been proposed regarding the transfer of mitochondrial DNA. Microvesicles and tunneling membrane nanotubes have been shown to be involved in the transfer of mitochondria between cells. An in vitro study suggested that cell fusion is also a possible mechanism that participates in the transfer of mitochondria [154]. Furthermore, shuttling of mitochondrial between cells, which form junctions not requiring complete fusion of cells, cannot be eliminated. In conditions of compact tissue architecture, nanotubes, or even direct cell to cell connections, which implicate lose junctions centered on, for instance, cytoskeletal and hemi-connexins structures are likely. In some systems (in vitro), connexin 43 has been reported to occur at the junction with the nanotubes [152,157]. Furthermore, large microvesicles that can have entire mitochondria with normal respiratory roles have also been demonstrated to be produced through astrocytes during cell culture, and when administrated into ischemic brain damaged mice, these microvesicles associated with injured neurons [153], indicating the microvesicles’ potential to mediate uptake of mitochondria in the mice brain. Various situations would be possible depending on whether these vesicles were endocytosed, and then release the mitochondria into cell cytoplasm rather than entering the pathway of lysosomes, or whether they combine with the cell membrane leaking their mitochondrial contents into the cell cytoplasm. In both the situations, cytosolic mitochondrial DNA level is enhanced, which is recognized by the nucleic acid sensors in the cytoplasm. This enhancement of mitochondrial DNA in the cytoplasm could trigger the innate immune system [71,72]. Overall, the intercellular transfer of mitochondrial DNA and its implications in the innate anti-microbial immunity has rarely been studied, and future research should focus on this essential physiological phenomenon to improve our knowledge regarding the innate immune system. 

## 5. Conclusions

To date, remarkable progress has been made in the last several years to describe the biological mechanisms by which the innate immune system recognizes microbial pathogens and integrates signals from pathogen associated molecular patterns to mount a precise immune response [158,159,160]. Recently it has been shown that under cellular damage and stress conditions (e.g., microbial infections), mitochondrial DNA is generally released into cytoplasm instead of genomic DNA. This cytosolic mitochondrial DNA plays a crucial biological role in limiting microbial infection and is also involved in the development of various inflammatory diseases by stimulating signaling cascades. Many studies provided evidence regarding the activation of NLRP3, TLR9, and STING by mitochondrial DNA, indicating its implication in the regulation of innate immune signaling [45,49,58,62]. Many interesting questions remain unanswered regarding the mitochondrial DNA and its biological role in innate immunity. For example, is there some other mitochondrial DNA signaling pathway that exists, which remains undiscovered? What are the specific biological mechanisms, which are implicated in the process of mitochondrial DNA being leaked from the mitochondria? One study reported that releases of mitochondrial DNA is mediated by mitochondrial permeability transition based on sensitivity to cyclosporine A, which is plausible if mitochondrial permeability transition is followed by osmotic swelling and rupture of the mitochondrial membrane [57]. A recent study suggested that the hydrolysis of the mitochondrial membrane by secreted phospholipase A2 IIA (sPLA2-IIA) produces inflammatory mediators (e.g., mitochondrial DNA and lysophospholipids) that induce activation of leukocytes, leading to different inflammatory responses [161]. Furthermore, some studies also demonstrate that various viral proteins are responsible for the release of mitochondrial DNA in the cytosol. There is an interesting question require to deepen our knowledge regarding the mitochondrial DNA further. For example, during *Mycobacterium tuberculosis* infection, because of mitochondrial damage, DNA is released from mitochondria that is considered is an important source to bind with cGAS [73]. Furthermore, *M. tuberculosis* DNA is also a prime source for the activation of this signaling cascade [162]. Considering the mitochondrial DNA as a source for activation of these signaling pathways during microbial infection; if so, does it affect both type I IFN expression and selective autophagy? The contribution of mitochondrial DNA to bacterial autophagy is especially interesting given the similarity of this signaling pathway to mitophagy, and the fact that the mitochondrion itself was once a bacterium [73,163]. More studies and experimentation are required to further understand the results of cellular damage and the participation of host mitochondrial DNA during microbial infection. 

Furthermore, another interesting question is to understand the mechanism by which DNA sensing molecules can potentiate various outcomes of microbial infection. For example, in the case of *M. tuberculosis*, cGAS, STING, and TBK1 are all needed for both autophagosomal targeting and type I IFN expression. How can these identical molecules be simultaneously implicated in such various signaling pathways? One possibility is that the post-translational modifications mediates protein–protein interactions that favor associations at either the in complexes or phagosomal membrane that enhances expression of type I IFN. Other possibility is that TBK1 plays a privileged biological role in polarizing DNA, recognizing outcomes based on its ability to phosphorylate various substrates, such as IRF3. Identification and characterization of these substrates may be critical to understanding how mitochondrial DNA recognizing potentiates both type I IFN expression and autophagy.
